# Protoporphyrin IX Binds to Iron(II)-Loaded and to Zinc-Loaded Human Frataxin

**DOI:** 10.3390/life13010222

**Published:** 2023-01-12

**Authors:** Ganeko Bernardo-Seisdedos, Andreas Schedlbauer, Tania Pereira-Ortuzar, José M. Mato, Oscar Millet

**Affiliations:** 1 ATLAS Molecular Pharma, Bizkaia Science and Technology Park, 48160 Derio, Spain; 2 Precision Medicine and Metabolism Laboratory, CIC bioGUNE, Basque Research and Technology Alliance (BRTA), Bizkaia Science and Technology Park, 48160 Derio, Spain; 3 Biomedical Research Network on Hepatic and Digestive Diseases (CIBEREHD), Instituto de Salud Carlos III, 28029 Madrid, Spain

**Keywords:** frataxin, iron metabolism, heme biosynthesis, iron-sulfur clusters, ferrochelatase, Friedreich’s ataxia, NMR spectroscopy

## Abstract

(1) Background: Human frataxin is an iron binding protein that participates in the biogenesis of iron sulfur clusters and enhances ferrochelatase activity. While frataxin association to other proteins has been extensively characterized up to the structural level, much less is known about the putative capacity of frataxin to interact with functionally related metabolites. In turn, current knowledge about frataxin’s capacity to coordinate metal ions is limited to iron (II and III); (2) Methods: here, we used NMR spectroscopy, Molecular Dynamics, and Docking approaches to demonstrate new roles of frataxin; (3) Results: We demonstrate that frataxin also binds Zn^2+^ in a structurally similar way to Fe^2+^, but with lower affinity. In turn, both Fe^2+^-loaded and Zn^2+^-loaded frataxins specifically associate to protoporphyrin IX with micromolar affinity, while apo-frataxin does not bind to the porphyrin. Protoporphyrin IX association to metal-loaded frataxin shares the binding epitope with ferrochelatase; and (4) Conclusions: these findings expand the plethora of relevant molecular targets for frataxin and may help to elucidate the yet unknown different roles that this protein exerts in iron regulation and metabolism.

## 1. Introduction

Frataxin (FXN) is a single-domain protein of 155 amino acids in its mature form (human homolog) [1], showing a large structural conservation among species [2,3,4,5]. FXN is nuclear-encoded with mitochondrial localization [6], is expressed mainly at the tissues that are dependent on oxidative respiration [7], and it plays a key role in intracellular iron homeostasis and metal ion disposition [8,9]. An insufficient amount of FXN leads to the rare early-onset disease Friedreich’s ataxia (FA), characterized by sensory and cerebellar ataxia, among other symptoms [10]. At the molecular level, FA produces the accumulation of iron and other metals in serum [11] or cardiomyocytes [12] and the dysregulation of many genes and metabolites, with the elevation of intracellular levels of protoporphyrin IX (PPIX), despite the downregulation of coproporphyrin oxidase (and ferrochelatase, FCH) [13].

The role of FXN in iron homeostasis relies on its capacity to bind Fe^2+^ [14,15] and, to a lesser extent, to bind Fe^3+^ [16,17]. Consistently, pathogenic mutations found in FA patients show a limited capacity to retain iron atoms [17,18]. The iron/protein interaction is mainly mediated by the surface carboxylates in the region and composed of the first α-helix and β-strand elements [19] (here referred to as the canonical binding region). The binding stoichiometry of iron to human FXN is not exactly stablished but it is large, with FXN binding between six and seven ions at low micromolar affinity (K_D_ ≈ 50 μM) [15], suggesting a metallochaperone role for the protein. Recent structural elucidations in human and yeast homologs have established a constitutive role for FXN in the formation of iron sulfur clusters (ISC) [20], by binding to the cysteine desulfurase of the iron sulfur pathway protein cluster [21]. The FXN-loaded complex structure shows that the protein facilitates ISC production through the stabilization of key loop conformations in one of the cluster constituents [21,22].

Alternatively, many biochemical studies in human and yeast FXN demonstrate that it also binds tightly to FCH, the last enzyme in the heme group biosynthesis [2,23,24]. Such interaction occurs at the canonical binding region in FXN and a docking simulation locates FXN opposite to the active site of the enzyme [23]. At the functional level, FXN binding to FCH is not essential but it enhances the production of heme [13,25]. The binding of other metals to FXN has been indirectly inferred but never demonstrated. In an FXN knock-out cellular model with reduced levels of FCH, it was shown that cellular heme production is modulated by the insertion of zinc instead of iron into the porphyrin ring [26], which is consistent with the FCH ability to chelate zinc [27]. Here, we demonstrate that FXN binds the diamagnetic ion Zn^2+^, preserving the protein’s binding epitope with the paramagnetic Fe^2+^.

These two identified functions are complementary and suggest that FXN may be a central element for iron distribution between heme and ISC biosynthesis, an interplay that is not totally understood. Supporting this idea, a model of mitochondrial heme metabolism protein complex has been proposed in which FCH and ISC production are structurally coupled [28]. In this context, the binding of other metabolites to FXN could help in understanding the mitochondrial regulation of frataxin. It has been reported that CyaY from *Vibrio cholerae*, an FXN homolog, is able to bind heme with nanomolar affinity and a putative regulatory role has been suggested [3]. However, no studies in human FXN binding to metabolites have been performed. Here, we present experimental evidence to demonstrate that both, Fe^2+^- and Zn^2+^-loaded FXN, are able to bind to PPIX. Such binding competes (but it does not abrogate) the interaction between FCH and FXN.

## 2. Materials and Methods

### 2.1. Reagents

Protoporphyrin IX (PPIX, M_w_ = 562.66 g/mol) was purchased from Frontier Scientific (Cat# C654-3, CAS 14643-66-4). A 1 mM stock solution of PPIX in ethanolic KOH was prepared by dissolving 5.6 mg in 2.5 mL 100 mM KOH by vigorous vortexing before adding 2.5 mL of absolute ethanol. Solution can be stored at −20 °C for a couple of months.

### 2.2. Protein Expression and Purification

Expression and purification of the metallochaperone human Frataxin. The recombinant plasmid *FXN_pGS21a* (purchased from Genescript) encoding for residue D91 to A210 of the wild type human frataxin protein bearing a N-terminal 6xHis-GST tag was transformed into *E. coli* BL21 cells, and expressed under control of the T7 promoter at 30 °C for about 16 h in minimal M9 media (24 mM Na_2_HPO_4_, 11 mM KH_2_PO_4_, 4.3 mM NaCl, 2 mM MgSO_4_, 0.1 mM CaCl_2_, 200 mg/L thiaminehydrochloride, 3 g/L glucose, 100 mg/L kanamycin, and 1 g/L ^15^NH_4_Cl (Sigma-Aldrich, Burlington, MA, USA) as the only nitrogen source supplemented with trace metal mix composition according to F. Studier [29] for uniform ^15^N labeling.

The harvested cells were frozen at −80 °C, resuspended in lysis buffer (20 mM Tris pH = 8, 120 mM NaCl, 2 mM imidazole, 10% glycerol, 0.1% triton-X100, and one tablet of cOmplete™ EDTA-free protease inhibitor cocktail), incubated for about 30 min on ice prior completing cell lysis by ultrasonication. The cell debris was removed by ultracentrifugation at 60 kg. The soluble fraction was passed through a 0.22 μm filter and loaded onto 3 mL cobalt-charged NTA agarose column equilibrated with lysis buffer before extensive cleaning with washing buffer (20 mM Tris pH = 8, 120 mM NaCl, 2 mM imidazole, and 10% Glycerol) for at least 20 column volumes (CV). The protein was eluted in one step applying 200 mM imidazole in the washing buffer. For subsequent imidazole removal the buffer of the pooled fractions was exchanged to 20 mM Tris pH = 8, 120 mM NaCl, and 10% glycerol using Sephadex™ G-25 desalting column. Afterwards, the 6xHis-GST tag was cleaved by incubation at room temperature for 6 h applying 2 units of thrombin per mg of target protein. The cleaved protein was separated from 6xHis-GST and uncleaved protein by a second affinity step before loading the sample on a Sd75/16/600 gel filtration column for final polishing. The apparent elution volume was 76.5 mL. The purity of the obtained protein (13.8 kDa) was monitored using SDS-PAGE (5–20% gradient gel) and the concentrations were determined spectrophotometrically (using a extinction coefficient ε of 26,930 cm^−1^M^−1^) and by a Bradford assay.

Expression and purification of active human ferrochelatase. The recombinant expression of a protein encompassing residue R65 to residue L423 of human ferrochelatase cloned into pET28a vector (purchased from Genescript) was achieved in Luria–Bertani media (containing 100 mg/L kanamycin) without any further supplements.

The harvested cells were frozen at −80 °C and resuspended in lysis buffer (50 mM MOPS-Tris pH = 8.0, 100 mM KCl, 1% sodium cholate, one tablet of cOmplete™ of EDTA-free protease inhibitor cocktail, 10% glycerol, and 100 μM TCEP) applying a ratio (*w*/*v*) buffer to wet cell pellet of 7:1. Then, the cell suspension was incubated on ice for about 30 min prior to ultrasonication. After clearance of the lysate by centrifugation at 60 kg for about one hour the supernatant was passed through a 0.22 μm filter and applied twice on a 3 mL cobalt charged HisTrap resin equilibrated with lysis buffer. The bound ferrochelatase protein was excessively washed with stabilization buffer (50 mM MOPS-Tris pH = 8.0, 100 mM KCl, 1% sodium-cholate, 10% glycerol, and 100 μM TCEP) for about 40 CV before performing a one-step elution with a buffer containing 250 mM imidazole. The pooled fractions were concentrated to max. 300 μM using Vivaspin device (cutoff 10 kDa) and applied on Sd200/16/600 gel filtration column equilibrated with washing buffer. The protein eluted as a homodimer (88 kDa) at 81 mL. Protein purity was verified by gradient SDS-PAGE (4.5–20%) and its concentration was determined spectrophotometrically using a molar extinction coefficient ε for the ferrochelatase homodimer ε of 96.720 M^−1^cm^−1^ and a molecular mass of 88 kDa.

Functional activity of the enzyme was verified using zinc as a metal substrate allowing to conduct the activity assay under aerobic conditions (Figure S1). Analysis of metalloporphyrin product was conducted by high-performance liquid chromatography (HPLC). For that purpose, 20 μL of ferrochelatase (final concentration 4 μM) in stabilization buffer were added to 170 μL of protoporphyrin IX dissolved in Tris/palmitate/Tween 20 buffer (100 mM Tris-HCl pH = 8.0, 1 mM palmitic acid, and 0.3% Tween-20, sonicated in a water bath prior usage under light protection for two minutes). The enzymatic reaction was started adding 10 μL of 2 mM zinc acetate (final concentration 100 μM) and incubated under light protection at 37 °C for about 30 min. Termination of the reaction was achieved by adding 500 µL of 3:7 (*v*/*v*) dimethyl sulfoxide/methanol mixture and short vortexing followed by centrifugation at 14,000× *g* for 10 min at 4 °C. An amount of 50 μL of the supernatant were applied to a LiChrospher 100 RP-18 (5 mm) column performing the separation at 29 °C with a gradient of 90% methanol (*v*/*v*) in 1 M ammonium acetate (pH = 5.16), and methanol as eluent. The chromatogram was monitored by fluorometric detection (excitation 405 nm; emission 620 nm).

### 2.3. NMR Spectroscopy

NMR experiments were performed with a Bruker Avance III 800 MHz spectrometer equipped with a triple resonance ^1^H, ^13^C, ^15^N-cryoprobe (TCI). For Fe^2+^ and/or PPIX titrations, two-dimensional ^1^H-^15^N SOFAST-HMQC spectra (1 h duration) were recorded at 298 K, using ^15^N-labelled FXN protein at 30 µM and varying Fe^2+^ and/or PPIX quantity so that the molar ratio was 1:[0,1,2,3,4,5]:[0,1,2,3,4,5] (FXN:Fe^2+^:PPIX). For Fe^2+^ and PPIX titrations, two-dimensional CON spectra (14 h duration) were recorded at 298 K using ^13^C-^15^N-labelled FXN protein at 255 µM and varying Fe^2+^ and/or PIX quantity so that the molar ratio was 1:[0,1,3]:[0,1] (FXN:Fe^2+^:PPIX). Urea (10 mM) was added for the monitorization of the non-specific Fe^2+^ paramagnetic effect in the CON experiments: the intensity of each peak is normalized by the peak intensity for the corresponding urea peak (162;104 ppm). For titrations performed with Zn^2+^ and/or PPIX, two-dimensional ^1^H-^15^N SOFAST-HMQC spectra (1 h duration) were recorded at 298 K using ^15^N-labelled FXN protein at 100 µM and varying Zn^2+^ and/or PPIX quantity so that the molar ratio was 1:[0,1,2,3,4,5]:[0,1,2,3,4,5] (FXN:Zn^2+^:PPIX). All the samples were prepared under strict anaerobic conditions.

Spectral peak assignment was performed using CCPNMR software using reference chemical shift values obtained from the Biological Magnetic Resonance Data Bank with code BMRB: 4342 (PDB:1LY7) [1]. Chemical shift and peak intensity values were extracted for each titration point and analysed with in-house built MatLab scripts. The chemical shift perturbation (CSP) was determined according to the following equation:

$$\mathrm{CSP}=\sqrt{0.1*\delta_{N}^{2}+\delta_{H}^{2}}$$

were *δ_N_* and *δ_H_* correspond to the nitrogen and proton chemical shifts, respectively. Only values that differed in more than one standard deviation from the average value were considered as significant. Plots were originally prepared in xmgrace (https://plasma-gate.weizmann.ac.il/Grace/ accessed on 25 September 2022) and then edited in inkscape.

The EC_50_ calculation was performed using a collective fitting to the Hill–Langmuir equation defined as:

$$\Delta \delta =\Delta \delta_{max}*\frac{L^{n}}{EC_{50}+L^{n}}$$

where Δ*δ_max_* is the maximum CSP. The CSP data was averaged from the following 30 residues: 93, 95, 98, 100, 104, 105, 106, 112, 114, 116, 120, 129, 136, 140, 144, 152, 153, 169, 173, 176, 184, 185, 188, 190, 191, 195, 197, 199, 207, 208. The result of the fitting estimated the hill coefficient on *n* = 1.01, Δ*δ_max_* to 1.7895, and an EC_50_ on 140.23 µM.

### 2.4. Molecular Dynamics Simulations and Ligand Docking

Molecular dynamic (MD) simulations were carried out as previously described [30,31]. The structure of human frataxin (PDB: 1LY7, model1) [1] was used for all the simulations. The atomic structure was then solvated in a rectangular water box of dimensions 54.4 × 72.1 × 57.5 Å^3^ using the Solvate plug-in of VMD [32]. Na^+^ and Cl^−^ ions were added to the system to achieve charge neutralization up to a final concentration of 120 mM. Moreover, 30 Zn^2+^ ions were loaded to the system. The resulting system comprised ~21.000 atoms and the simulation was run for 120 ns. The CHARMM 36 force field was employed to describe the molecular mechanics [33]. The TIP3P model was used for water. The standard CHARMM parameters were used for ions. NAMD version 2.10 was used for all calculations. Atomic coordinates were collected every 20.000 steps. During this trajectory, an equilibrated atomic structure was selected. The MD trajectories were analyzed using VMD.

Ligand docking simulation were accomplished using the open-source programs for AutoDock Vina and AutoDock4.2 in conjunction with AutoDock Tools (ADT) [34]. For Fe^2+^-loaded FXN and PPIX docking, 1LY7 pdb file was used as protein receptor [1] whereas for Zn^2+^-loaded FXN and PPIX docking a MD frame at 120 ns was used as receptor. In both cases, the resulting PPIX-induced CSPs were used as restraints for the docking process. Protoporphyrin SMILE code was found in PubChem database. Structure prediction, optimization, and refinement was achieved through minimization by applying CGs with a united force field by employing 25 × 10^6^ steps using Open Babel package (v 2.4.0). Receptor and ligand structures were adapted to AutoDock format using ADT tools, adding partial charges and atom types to the PDB format. Docking was performed adding flexibility to metal-loaded FXN residues that showed CSP upon PPIX binding in NMR experimental conditions. Finally, the Lamarckian geometric algorithm methodology was selected in the docking parameter.

## 3. Results

### 3.1. Iron(II) Titration to FXN as Monitored by NMR Spectroscopy

As already mentioned, a significant amount of experimental evidence supports the idea the FXN binds Fe^2+^ ions [14,15,17,19,24]. Fluorescence experiments demonstrate that FXN binds between six and seven ions simultaneously, with an average affinity of 55 µM [15]. Complementarily, NMR experiments under sub-stoichiometric conditions of iron (a limitation due to protein aggregation and non-specific intensity losses due to paramagnetic relaxation) have identified the first α-helix and β-strands of the protein as the canonical binding region for the ferrous ions, both in human and in yeast FXN [2,14,17]. However, this binding epitope is not large enough to accommodate all the expected ions. In fact, published analyses only considered chemical shift perturbations (CSP) as a reporter for interaction, but Fe^2+^ binding will also induce a residue-specific line-broadening due to paramagnetic relaxation, which may ultimately jeopardize the monitoring of additional CSP.

**Figure 1:**
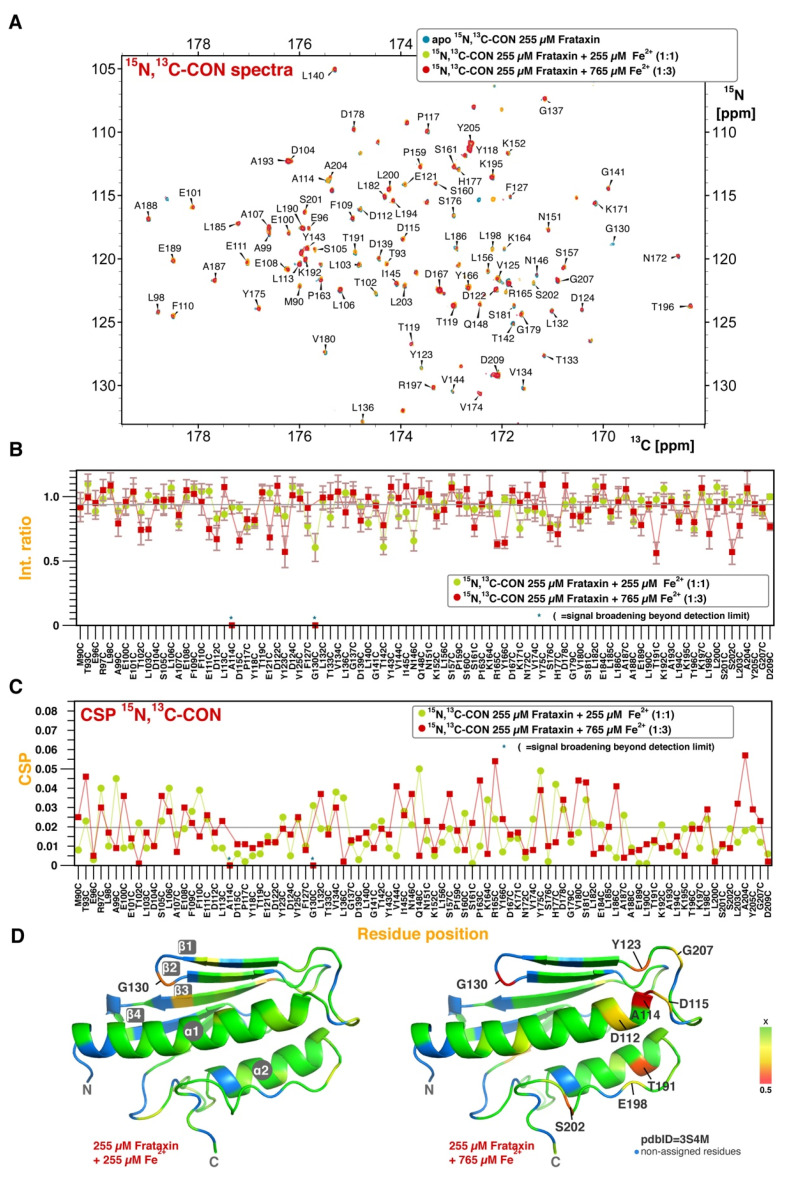
Fe^2+^ binding to FXN. (**A**) overlap of three different ^15^N,^13^C-CON spectra (as shown in the legend), showing the peak assignments for the backbone amide groups. (**B**) Intensity ratio normalized to the urea signal as a function of the amino acid for two different experimental conditions, as indicated in the legend. (**C**) Chemical shift perturbation (CSP) as a function of the amino acid for the same dataset. (**D**) Structural representation of the intensity variations at 1 equivalent (**left**) and 3 equivalents (**right**) of FeCl_2_. The color code reflects the intensity variations and it is proportional to the standard deviation scale from the average value, as indicated in the bar legend (x meaning average value). The gray lines correspond to the average values for all the residues.

The CON experiment sequentially connects amide ^15^N resonances using chemical shift matching with the preceding ^13^C-carbonyl nuclei in the protein backbone [35]. CON is less sensitive to CSP but, since this pulse sequence does not rely on proton magnetization, it is much less affected by paramagnetic relaxation. Moreover, CON is also devoid of chemical exchange with the solvent, so an internal reference can be added (urea, see Section 2) to quantitatively integrate the signal intensity as a surrogate reporter for metal binding. Figure 1A shows the assigned CON spectrum for FXN at 298 K, showing the typical excellent chemical shift dispersion obtained with this experiment, with no signal overlap. We have used CON to titrate the effect of Fe^2+^ on FXN (FeCl_2_, one to three equivalents, Figure 1A,B). Unfortunately, the use of larger amounts of iron results in the aggregation of the protein, as previously reported [36]. As expected, the peak intensity is much less perturbed upon iron addition in the CON compared with the TROSY-HSQC (Figure S2), enabling an accurate monitoring of the CSP for most of the peaks in the spectrum.

The CSP perturbation profile (Figure 1B), the titrant-induced intensity changes (Figure 1C), and the CSP location in the structure (Figure 1D) are consistent with the reported canonical binding region as the main epitope for the ferrous ion association, but they also reveal additional secondary binding sites for iron, ultimately involving many residues throughout the protein (i.e., region Y143-A187). This binding scenario is consistent with the large number of aspartic and glutamic residues in FXN, distributed across all the secondary structural elements [1] and it also provides a structural rationale for the elevated number of ferrous ions simultaneously coordinated by the protein [15]. Due to the sub-stoichiometric conditions and assuming a similar affinity to the different sites, we hypothesize that iron exchange between sites is mainly responsible for the signal intensity losses observed in the secondary binding sites (i.e., D115-D124 or T191-A204, Figure 1C).

### 3.2. Human FXN Binds Zn^2+^ in an Isosteric Mode as Compared to Fe^2+^

**Figure 2:**
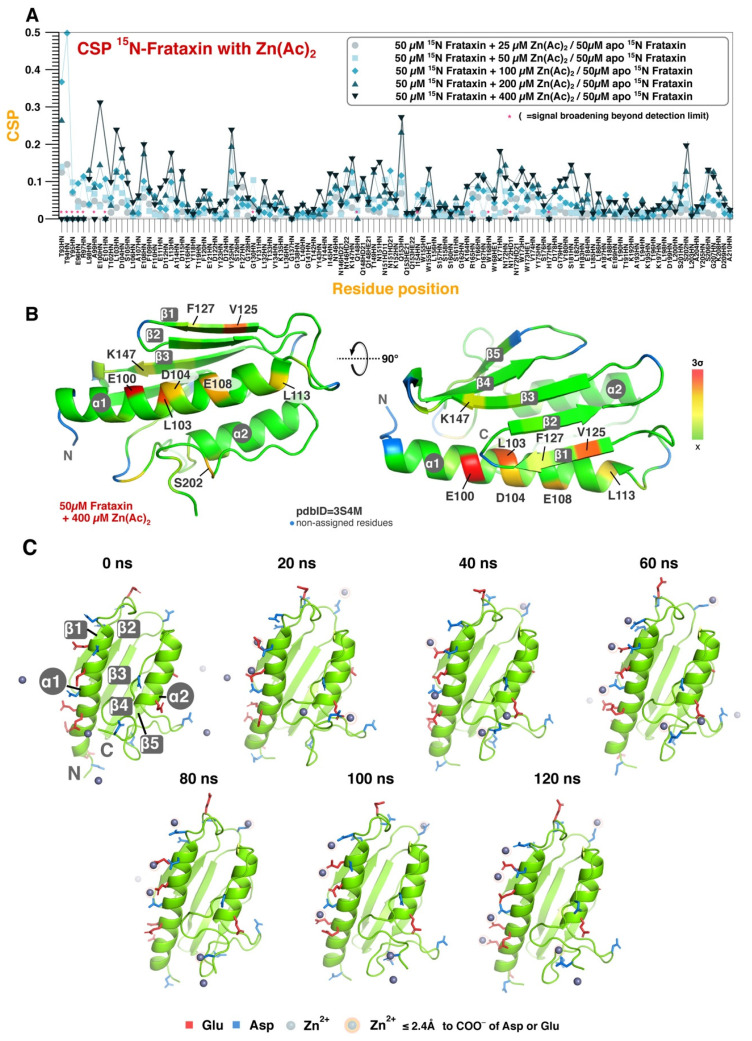
Zn^2+^ binding to FXN. (**A**) FXN amino acid CSP as a function of the Zn(Ac)_2_ concentration, as indicated by the color/symbol code. Titration was conducted in the absence of 1% cholate. (**B**) Structural representation of the CSP values at 8 equivalents of Zn(Ac)_2_ and in two different orientations. The color code is proportional to the standard deviation scale from the average value, as indicated in the bar legend (x meaning average value). (**C**) MD snapshots at the indicated times with the position of the nearby Zn^2+^ ions also displayed. Glowing spheres refer to the metal ions that are effectively coordinated to the surface of FXN. Relevant residues (i.e., Asp and Glu) are color coded, as indicated.

We next explored the capacity of FXN to bind other divalent ions, such as Zn^2+^, using NMR spectroscopy. Apart from the potential functional implication, Zn^2+^ is advantageous for these studies since it is diamagnetic and it produces no specific nor unspecific broadening in the spectrum due to paramagnetic relaxation. This enabled the possibility of exploring the binding of Zn^2+^ to FXN at conditions where the metal is in excess. Figure 2A shows the SOFAST-HMQC-monitored CSPs of FXN when titrated with up to eight equivalents of Zn^2+^ (ZnCl_2_) at 298 K. No aggregation nor protein precipitation was observed upon ligand addition. FXN effectively coordinates the metal ions and CSPs are observed throughout the protein, proportional to the ZnCl_2_ concentration. No indication of CSP saturation is observed at the last titration point and the intensity of many peaks corresponding to residues distributed throughout the protein decreased as a function of the titrant concentration (data not shown). In the absence of paramagnetic relaxation, such intensity decays must be attributed to the chemical exchange of ions within partially saturated sites, supporting the idea that Zn^2+^ binds weaker to FXN than Fe^2+^.

The Zn^2+^-induced CSP profile offers an unprecedented opportunity to investigate the metal binding to FXN. CSPs are observed throughout the protein, but at unequal strength, with the canonical binding site for iron (i.e., the first α-helix and the first two β-strands of the protein) being the most intense perturbation. However, other changes are also observed and Zn^2+^ coordination may occur at the surroundings of aspartic and glutamic residues, as expected, but also close to glutamine and asparagine residues, particularly in the region N146-Q153. Remarkably, the structural distribution of the Zn^2+^-induced CSPs (Figure 2B) agrees very well with the reported changes for association to Fe^2+^ [23] but also with the extended binding sites identified from the CON experiments (Figure 2A,B). Divalent zinc and iron have similar ionic radius and electronic distributions, explaining why they associate in an isosteric mode to the protein. Finally, cholate is a common cosolute of FECH (vide infra), which is often used in protein solubilization, and it may bind to non-polar patches. An equivalent zinc titration on FXN but in the presence of 1% cholate results in very similar CSP changes, except for the N-terminal region (L98-T102) (Figure S3), reinforcing the idea that Zn^2+^ binds primordially to negatively charged and/or polar residues.

We then obtained a structural model for Zn^2+^ association to FXN based on an MD simulation in water. During the simulation, Zn^2+^ ions bind to different regions of the protein at different time points, and they remain coordinated to the hydration layer of FXN for the rest of the simulation (120 ns). Remarkably, at the end of the simulation up to seven different Zn^2+^ ions can be observed directly interacting with residues E96, D104, E111, D115, D139, D209, and A210 (Figure 2C). Most of the residues (glutamate and aspartate) possess polar charged sidechains that are prone to interact with divalent ions. The C-terminus carboxylic group of A210 residue also could interact with divalent ions, as expected. Consistently, these residues also result in measurable CSPs in the titrations performed with Zn^2+^ or Fe^2+^. Interestingly, the MD simulation is able to clinch exchange events of Zn^2+^ ions between different binding sites, providing a rationale for the intensity losses observed in the NMR titrations.

### 3.3. PPIX Binds to Iron Loaded or to Zinc Loaded FXN

Since FXN binds to FCH and enhances its enzyme activity [2,4,23,24], the possibility of PPIX binding directly to FXN is conceivable, either as a functional association during catalysis or as a regulatory mechanism to manage the pool of available substrate and iron resources. Here, we have explored PPIX binding to apo-FXN or to holo-FXN (i.e., FXN loaded with one equivalent of Fe^2+^ or with four equivalents of Zn^2+^) at 298 K, using NMR spectroscopy (^1^H-^15^N SOFAST-HMQC).

**Figure 3:**
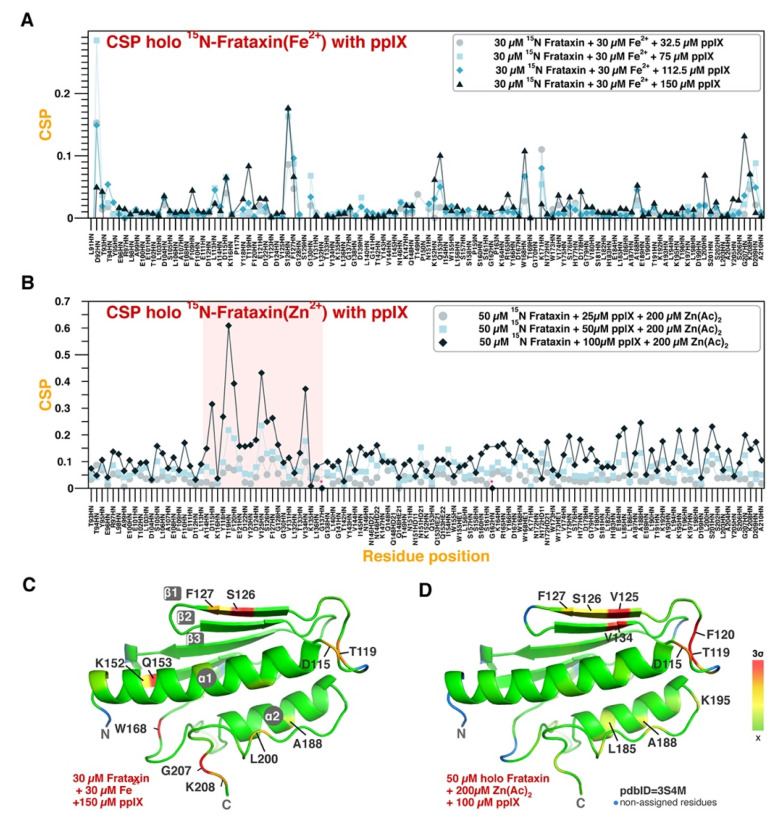
PPIX binding to Fe^2+^-loaded and Zn^2+^-loaded FXN. (**A**,**B**) FXN amino acid CSP as a function of Fe^2+^ concentration (**A**) or Zn^2+^ concentration (**B**), as indicated by the colored symbols in the legend. The pink highlighted area (in (**B**)) corresponds to the canonical binding site for iron. Titration was conducted in the absence of 1% cholate. (**C**,**D**) Structural representation of the CSP values at 1:5 equivalents of Fe^2+^:PPIX (**C**) and 4:2 equivalents of Zn^2+^:PPIX (**D**) of FeCl_2_. In (**C**,**D**) the color code is proportional to the standard deviation scale from the average value, as indicated in the bar legend (x meaning average value). Asterisk indicates signal broadening beyond detection limit.

No changes in chemical shift nor peak intensity changes could be observed when PPIX is titrated over apo-FXN (Figure S4). Instead, an equivalent titration targeting holo-FXN (either Fe^2+^-loaded or Zn^2+^-loaded) results in significant CSPs that are proportional to the titrant concentration (Figure 3A,B for Fe^2+^ and Zn^2+^, respectively). Structural mapping of the CSPs is shown for Fe^2+^-loaded and Zn^2+^-loaded FXN in Figure 3C,D, respectively. In holo-FXN, PPIX binds to the canonical binding region for the metal (Fe^2+^ or Zn^2+^), but it also perturbs other unrelated regions of the protein (mainly Q148-I154, W168, and T196-L200). We hypothesize that PPIX interacts with the loaded metal ions: stronger PPIX-induced CSPs are observed when targeting Zn^2+^-loaded FXN as compared to Fe^2+^-loaded FXN, but we attribute this effect to the higher metal loading load on the Zn^2+^-loaded FXN (four equivalents).

**Figure 4:**
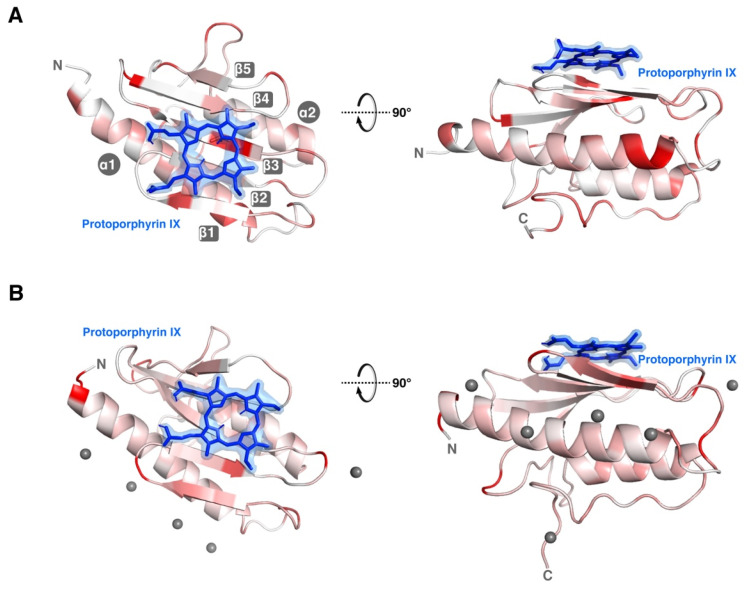
Structural model of FXN:PPIX binding in presence of Fe^2+^ or Zn^2+^ metal ions. (**A**) Docking model of PPIX in FXN where the Fe^2+^-induced CSPs are red color coded. (**B**) Docking model of PPIX in FXN where the Zn^2+^-induced CSPs are red color coded and with the metal ions’ location from the MD simulation.

The resulting CSPs induced by PPIX on the metal-loaded FXN (both Zn^2+^ and Fe^2+^) have been used as restrains to obtain docking models of PPIX binding to FXN (Figure 4A for Fe^2+^ and Figure 4B for Zn^2+^). Both docking models render very similar results, with PPIX stacking in the surface constituted by strands 1 to 3, also partially occupying the canonical iron binding site. Furthermore, a semi-quantitative analysis of the CSP indicates that the affinity constant falls in the micromolar range (EC_50_ ≈ 140 µM) (Figure S5). An accurate estimation of the K_D_ is precluded by the impossibility of saturating FXN with PPIX nor with metal ions.

### 3.4. FXN Has the Same Binding Epitope for FCH and PPIX

**Figure 5:**
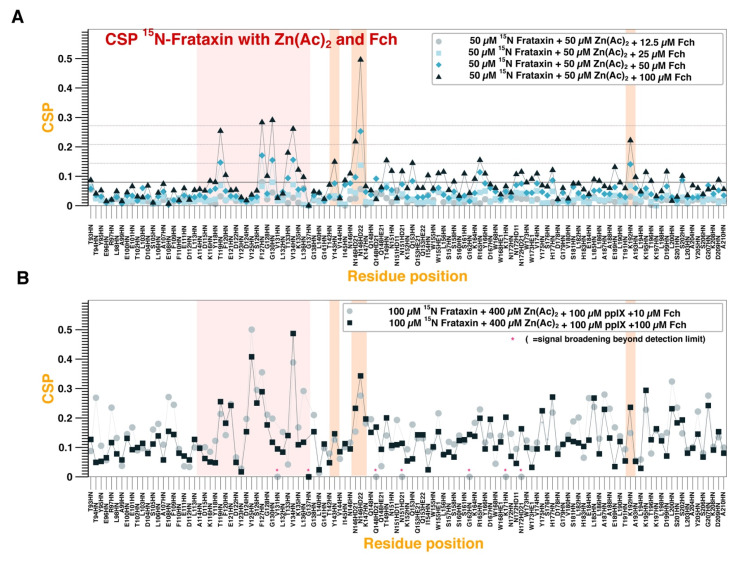
FXN binding to FCH in the presence/absence of PPIX. (**A**,**B**) FXN amino acid CSP as a function of FCH concentration in the absence (**A**) or in the presence (**B**) of 1 equivalent of PPIX. The Zn^2+^ concentrations are indicated in the figure legends. Color bands indicate the binding epitopes for PPIX (pink) and FCH (pink + orange).

It is well known that Fe^2+^-loaded FXN is able to bind FCH at the canonical binding region for iron [23]. Here, we also demonstrate that FCH also binds to Zn^2+^-loaded FXN and the CSP profile is shown in Figure 5A as a function of FCH concentration. Such CSPs are accompanied by a pervasive decay of the peak intensities (Figure S6), due to the line-broadening induced by the large molecular weight complex. Remarkably, the binding mode is totally homologous to the one previously reported for the FCH interaction with iron-loaded FXN [23] (Figure 5A).

Because FCH and PPIX largely share a binding epitope for FXN (marked region in Figure 5A), we have reproduced the FCH titration to FXN in the presence of two equivalents of PPIX and up to two equivalents of FCH (Figure 5B). Importantly, the regions I145-K147 and, to a lower extent, T191-A193, are characteristic of the FCH association to FXN (Figure 5B). These CSPs are maintained in the presence of PPIX (Figure 5B), indicating that equimolar amounts of PPIX are not able to abrogate the FCH–FXN interaction, consistent with the high-affinity of FCH for the metallochaperone [24].

### 3.5. Limitations of the Study

This study has several limitations. Most of the titrations are incomplete due to aggregation issues. Moreover, NMR spectroscopy tends to overestimate the K_D_ values due to the high concentrations of the receptor required for the titrations. The CON experiment is particularly insensitive to CSP. MD simulations cannot be used to extract thermodynamic nor kinetic information of the binding events and the conditions used (number of equivalents and time) are not physiological. All the reported experiments are biophysical studies limited by the reductionist approach and many other intracellular components may also bind to FXN, altering the significance of the results reported here.

## 4. Discussion

Human frataxin is an important mediator of intracellular iron metabolism, with its deficiency leading to a severe rare disorder [9,10,13]. The assigned metallochaperone role for FXN requires that it binds several iron ions and many studies in human and yeast FXN have pinpointed a canonical binding site for Fe^2+^ [2,14,17,23,37]. Such binding epitope is mediated by aspartate and glutamate residues and it involves about one third of the protein’s surface. However, this binding site is insufficient to allocate all the expected ions that the protein must bind according to fluorescence titrations [15]. The use of the CON experiment, less sensitive to the Fe^2+^-induced paramagnetic broadening, suggests that the metal interaction with the protein’s surface extends beyond the canonical site, which is also consistent with the widespread distribution of charged acidic residues (i.e., Asp and Glu) along the surface.

Here we also demonstrate that human FXN also binds Zn^2+^ in a structurally homologous way compared with Fe^2+^, though with a lower affinity. Several studies suggest that this binding may be functionally relevant: FXN mutations that result in FA also cause an elevation of the intracellular zinc levels [8,12]. Moreover, zinc stabilizes the metabolon that biosynthesizes the iron sulfur clusters, a protein complex that is participated by human FXN by interacting with a structural zinc ion [20,21]. Finally, the equivalent binding mode between Fe^2+^ and Zn^2+^ suggests a synergistic mechanism and we hypothesize that, under stress situations of low iron, zinc loading to FXN may be a surrogate binder to maintain the functional role of the metallochaperone.

We also report experimental evidence that supports a micromolar association of PPIX to metal-loaded FXN, to our knowledge a totally unprecedented observation. PPIX is the substrate for FCH, which also binds Fe^2+^-loaded FXN through the canonical binding region [23], and also to Zn^2+^-loaded FXN at the same site (this work). PPIX shares a binding site with FCH for their association to FXN, but PPIX is not able to abrogate the enzyme binding, with our data being also compatible with the formation of a complex FXN(Fe^2+^)-PPIX-FCH. Indeed, this complex could rationalize the enhancement of the FCH activity observed in the presence of FXN. Alternatively, PPIX association to FXN may have a regulatory role to retain the iron in conditions of high erythropoiesis. In line with this observation, FXN deficiency results in elevated levels of intracellular PPIX, despite the downregulation of coproporphyrinogen oxidase [13]. It is important to emphasize that the accumulation of any metabolite from the heme biosynthesis pathway is always toxic [38,39] and PPIX levels must be maintained tightly regulated to avoid deleterious effects [40]. PPIX binding to FXN may well contribute to such regulation.

Finally, the new functionalities reported here for human FXN reinforce the dual role that the protein has in accelerating heme biosynthesis and contributing to the iron sulfur cluster formation, through a zinc-mediated interaction. The possibility of a mitochondrial heme metabolon that structurally clusters all the involved proteins [28,41] is in complete agreement with our observations.

## 5. Conclusions

Here, we have used NMR spectroscopy and molecular dynamics simulations to study the binding of zinc and iron(II) to FTX. The main result is that most charged residues, evenly distributed across the protein’s surface, weakly associate with the metal ions. Loading FXN with metal enables FXN binding to PPIX, which occurs at the same docking site as human FCH.

## Data Availability

Data will be available upon demand.

## Supplementary Materials

The following supporting information can be downloaded at: https://www.mdpi.com/article/10.3390/life13010222/s1, Figure S1. FECH enzyme activity assay. Figure S2. FXN intensity rations upon Fe^2+^ binding. Figure S3. Effect of cholate in Zn^2+^ binding to FXN. Figure S4. PPIX does not associate to apo-FXN. Figure S5. EC_50_ determination for PPIX association to Fe^2+^-loaded FXN. Figure S6. Zn^2+^-loaded FXN intensity ratio as a function of FCH.
