# Supplementary material for: Protoporphyrin IX Binds to Iron(II)-Loaded and to Zinc-Loaded Human Frataxin
Source: Life (Basel). 2023 Jan 12;13(1):222. doi: 10.3390/life13010222 (PMC9866752; doi:10.3390/life13010222)
Supplement: Supplementary file 1 [file life-13-00222-s001.zip › life-2131786-supplementary.pdf]

Supplementary Material for:

Protoporphyrin IX binds to iron(II)-loaded and to zinc-loaded human frataxin

Ganeko Bernardo-Seisdedos[1,2,#], Andreas Schedlbauer[2,#], Tania Pereira-Ortuzar[2], José M Mato[2,3] and Oscar Millet[1,2,3,*]

[1]ATLAS Molecular Pharma, Bizkaia Science and Technology Park, 48160 Derio, Spain.

[2]Precision Medicine and Metabolism Laboratory, CIC bioGUNE, Basque Research and Technology Alliance (BRTA), Bizkaia Science and Technology Park, 48160 Derio, Spain.

[3]Biomedical Research Network on Hepatic and Digestive Diseases (CIBEREHD), Instituto de Salud Carlos III, Madrid, Spain.

[#] Both authors contributed equally.

*To whom correspondence should be addressed: omillet@cicbiogune.es

Contents:

Figure S1. FECH enzyme activity assay.

Figure S2. FXN intensity rations upon Fe^{2+} binding.

Figure S3. Effect of cholate in Zn^{2+} binding to FXN.

Figure S4. PPIX does not associate to apo-FXN.

Figure S5. EC_{50} determination for PPIX association to Fe^{2+}-loaded FXN.

Figure S6. Zn^{2+}-loaded FXN intensity ratio as a function of FCH.

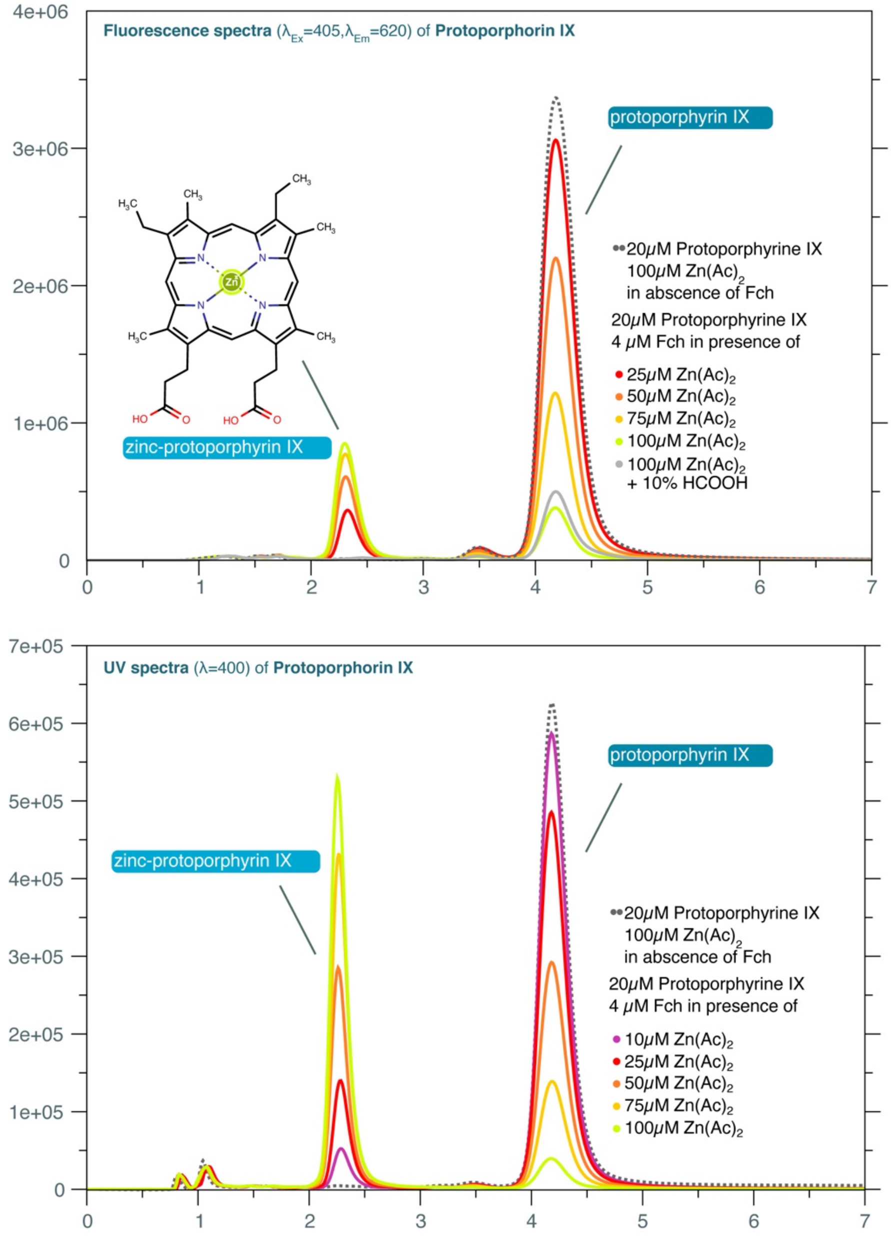

Figure S1. *FECH enzyme activity assay.* The chromatographic elucidation of Zn-PPIX and PPIX demomstrates FCH activity. In the absence of enzyme (dotted line) no Zn-PPIX is formed.

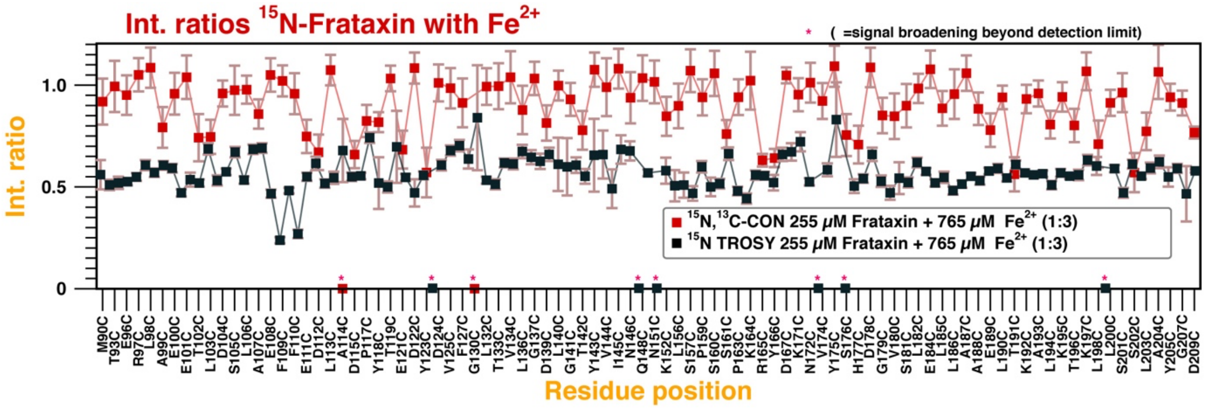

Figure S2. *FXN intensity rations upon Fe^{2+} binding*. Residue dependent comparison of the intensity ratios for the 15N-TROSY (black squares) with the CON experiment (red squares).

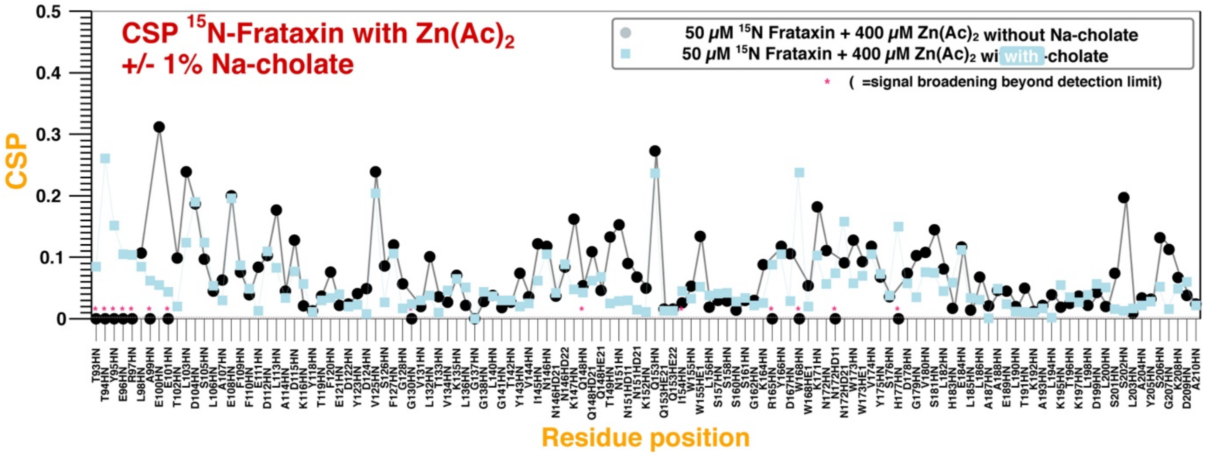

Figure S3. *Effect of cholate in Zn^{2+} binding to FXN*. Residue dependent comparison of the CSP induced by Zn^{2+} in the presence (blue squares) and in the absence (black circles) of 1% of sodium cholate.

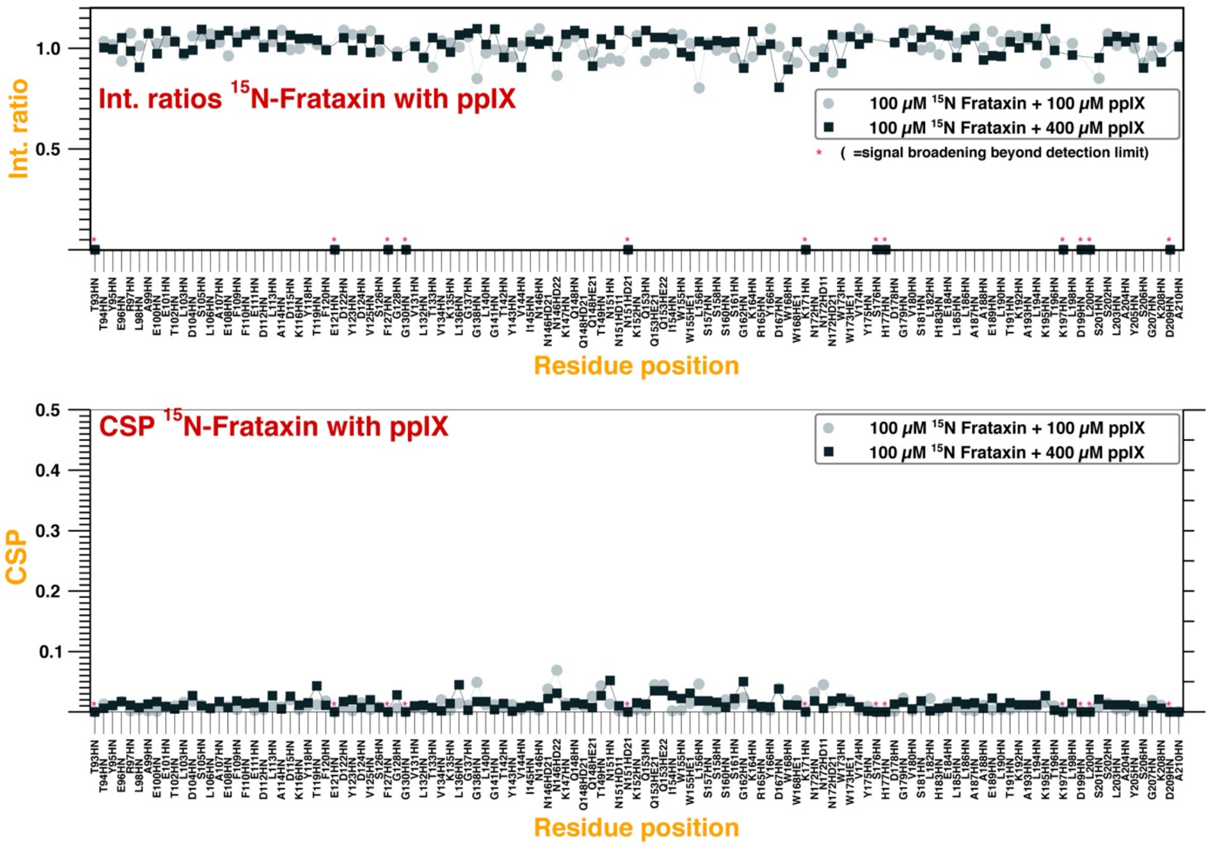

Figure S4. *PPIX does not associate to apo-FXN*. Changes in intensity (top) and CSPs (bottom) after the addition of 1 equivalent (gray circles) or 4 equivalents (black squares) of PPIX. Signals with a red asterisk showed a poor S/N (below 3) and they were not considered in the analysis.

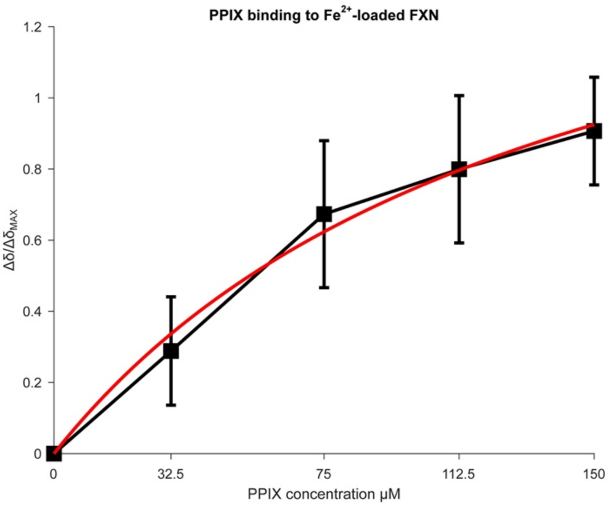

Figure S5. *EC_{50} determination for PPIX association to Fe^{2+}-loaded FXN*. Experimental data (black) and best fitting to the PPIX titration to Fe^{2+}-loaded (1 eq.) FXN (red curve).

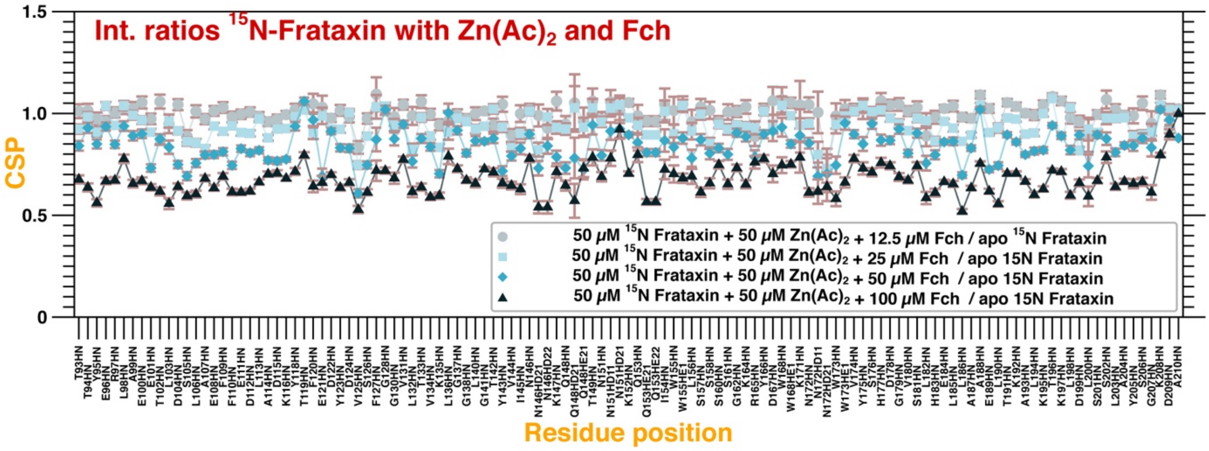

Figure S6. *Zn^{2+}-loaded FXN intensity ratio as a function of FCH*. The intensity ratios are coloured coded as indicated in the legend.
